# Organophosphate poisoning presenting with muscular weakness and abdominal pain- a case report

**DOI:** 10.1186/1756-0500-7-140

**Published:** 2014-03-12

**Authors:** Fazle Rabbi Chowdhury, Md Shafiqul Bari, MM Jahangir Alam, Md Mustafezur Rahman, Binayak Bhattacharjee, Junaid Abdul Qayyum, Md Sohel Mridha

**Affiliations:** 1 Department of Medicine, Sylhet MAG Osmani Medical College, Sylhet, Bangladesh

**Keywords:** Organophosphate, Poisoning, Pancreatitis, Intermediate syndrome

## Abstract

**Background:**

Organophosphate (OP) poisoning is the most common cause (27.64%) and has the highest death rate (13.88%) of poisoning in Bangladesh. It leads to three main syndromes notably acute cholinergic syndrome, intermediate syndrome, and delayed polyneuropathy. It rarely causes cardiac arrhythmia, pancreatitis and hepatic dysfunction. We present the case of a middle-aged Asian woman suffering from organophosphate poisoning with dual complications.

**Case presentation:**

A middle aged Asian woman with depression was brought to emergency attention after drinking of 60 milliliter of organophosphate insecticide in a suicidal attempt. She had vomiting, excessive retching, diarrhoea, miosis, hypersalivation and bilateral crepitation on chest during admission. After immediate resuscitation, atropinization was done and it required total of 36 milligram. The patient also received pralidoxime. While on maintenance, features of toxicity re-appeared and she again required atropine in bolus dose. On the fifth day of management she complained of generalized weakness, inability to control her neck and to sit or stand without support. But there was no respiratory muscle involvement and all deep tendon reflexes were normal. On the same day the patient also developed severe upper abdominal pain along with nausea and vomiting. Investigations revealed neutrophilic leucocytosis (30,000/cubic millimeter; 86%) with high serum lipase (770 Unit/Liter) and alanine transaminase (379 Unit/Liter) and low serum potassium (3.0 millimol/Liter). On the basis of above mentioned features organophosphate induced intermediate syndrome and pancreatitis was diagnosed. The patient recovered completely with appropriate management.

**Conclusion:**

Organophosphate poisonings causes up to 25% mortality worldwide. A major contributing factor for that are different complications. Awareness of these complications can reduce both mortality and morbidity. Early diagnosis of complications and timely therapeutic measures can improve prognosis.

## Background

Poisoning is a serious public health problem in Bangladesh comprising around 8-10% of overall mortality in medicine wards [1]. Organophosphate (OP) poisoning is the most common cause (27.64%) and has the highest death rate (13.88%) of poisoning in Bangladesh [2]. OP poisoning leads to three main syndromes: (1) acute cholinergic syndrome, (2) intermediate syndrome, and (3) OP induced delayed polyneuropathy [3]. Some other known complications of OP poisoning include cardiac arrhythmia, extrapyramidal features, pancreatitis and hepatic dysfunction. OP induced acute pancreatitis is a rare complication [4]. There are very few case reports on OP induced acute pancreatitis, yet it is believed that a large number of cases are under-reported. It is important to be aware of these complications for appropriate clinical management. In this write up we present a case of a woman with OP poisoning who presented with dual complications, both intermediate syndrome and acute pancreatitis.

## Case presentation

A 37-year-old Asian housewife was brought to the emergency about one and half an hour after drinking of one bottle (60 ml) of organophosphate insecticide in a suicidal attempt. She had previous psychiatric illness manifested as depression and occasional phobia following unemployment and since delivery of an autistic child. She had presented to us with vomiting, excessive retching and diarrhoea. She had miosis, hypersalivation and bilateral crepitation on chest. Gastric lavage was given immediately after admission. 2.4 mg (4 ampoules) atropine was started and the dose was doubled every 10 minutes until full atropinization. A total 60 ampoules (36 mg) atropine was required for atropinization and then patient was kept under maintenance dose of atropine in intravenous (IV) drip. Injectable Pralidoxime was also given for the first 24 hours. On third (3^rd^) day again the cholinergic symptoms re-appeared and re-atropinization with bolus dose was required. Complete blood count (CBC), electro cardiogram (ECG) and serum electrolyte on 3^rd^ day were normal. On fifth (5^th^) day of maintaining atropine therapy the patient complained of generalized weakness, inability to control the neck and to sit or stand without support. But there was no respiratory muscle involvement and all deep tendon reflexes were normal. On the same day the patient was also complaining of severe upper abdominal pain, nausea and vomiting. There was marked tenderness around the epigastrium, but the abdomen was soft with normal bowel sounds. Investigations at that time revealed (Table 1) white blood cell (WBC) count 30,000/cu mm with neutrophil count 86%, serum lipase 770 unit/Liter (U/L), serum alanine transaminase (ALT) 379 U/L and serum potassium 3.0 mmol/L. ECG and serum creatinine were normal. Ultra-sonogram of the hepato-billiary system (HBS) revealed swollen pancreas.

**Table 1:** Laboratory profile of the patient according to day

**Day**	**CBC	**ECG	**Serum electrolyte**	**Serum lipase**	Serum **ALT	**Serum creatinine**	**USG of HBS
**03**	Total count (TC)- 14000/cu mm with normal differentials	Normal	Normal	Not done	Not done	Not done	Not done
**05**	TC- 30,000/cu mm with Neutrophil 86%	Normal	Normal except serum K- 3.0 mmol/L	770 **U/L	379 **U/L	0.9 mg/dl	Swollen pancreas

**Complete blood count (CBC); Electrocardiogram (ECG); Alanine transaminase (ALT); Ultra sonogram of hepato-billiary System (USG of HBS); Unit/Liter (U/L).

On the basis of above mentioned clinical, biochemical and radiological findings the diagnosis of OP induced acute pancreatitis with intermediate syndrome was made. The patient was managed with I/V antibiotic, adequate analgesics and nasogastric feeding. A total 10 days of atropine therapy was required. After consultation with department of Physical Medicine, appropriate physiotherapy was started. We also consulted with psychiatry department and antidepressant drugs along with psychotherapy were given. The patient was discharged from hospital with good general condition and is now on regular follow-up with complete recovery of weakness.

## Discussion

Organophosphate compounds are possibly the most widely used insecticide in the world [5]. Intake of OP in a suicidal attempt is seen very often in Bangladesh as they are readily available. OP compounds phosphorylate the active site of acetyl cholinesterase, inactivating the enzyme causing accumulation acetylcholine which stimulates excessively nicotinic and muscarinic receptors producing widespread clinical symptoms like nausea, vomiting, diarrhea, urinary incontinence, blurring of vision, salivation, lacrimation, broncorrhoea, bradycardia, hypotension, muscle paralysis, fasciculation, confusion, seizures, coma and respiratory failure [5].

Intermediate syndrome is known as a syndrome of muscular paralysis occurring 24–96 hours following exposure, after resolution of acute cholinergic syndrome being treated with atropine [3]. Here weakness rapidly affects muscles of head and neck, proximal limbs and often the muscles of respiration causing ventilatory failure. The underlying mechanism of intermediate syndrome is not clearly understood, but it is generally believed to result from a persistent excess of acetylcholine at the neuromuscular junction [3]. In our patient muscles of neck and proximal limbs were involved sparing the muscles of respiration. OP induced acute pancreatitis is thought to result from prolonged hyper-stimulation of pancreatic acinar cells by acetylcholine and increased pressure in pancreatic ducts [6,7]. OP induced acute pancreatitis is believed to follow a subclinical and uneventful course [6]. Moreover the diagnosis of acute pancreatitis can be obscured by the systemic effects of OP toxicity. Though we deal with a large number of OP poisoned patients, there is no local data available regarding the frequency of high amylase or lipase levels and the incidence of acute pancreatitis in these patients. Few studies showed that raised amylase level was frequently seen in OP poisoned patients due to cholinergic stimulation to pancreas, but it is the raised lipase level which leads to the suspicion of acute pancreatitis [7]. Raised amylase level can also be found in other conditions like perforated peptic ulcer disease, intestinal ischaemia or ruptured ovarian cyst. In our patient we did only the serum lipase level considering it more specific which was found more than 2.5 times of normal. Neutrophilic leukocytosis and swollen pancreas both can be explained by the acute inflammation. Raised ALT may be due to OP induced hepatic dysfunction and or for acute pancreatitis itself. Hypokalaemia can be explained by diarrhea and vomiting.

## Conclusion

Worldwide studies suggest 3-25% mortality from OP poisoning [7]. Acute pancreatitis and intermediate syndrome are two grave complications of it. The incidence of intermediate syndrome may be as high as 80% and the syndrome has been considered a major contributing factor of OP related morbidity and mortality [5]. Mortality rate of hospitalized acute pancreatitis patients is between 5 and 10% in most series [6]. Awareness of these complications should prompt earlier investigations, because early diagnosis and timely therapeutic measures may improve the patient’s prognosis.

## Consent

Written informed consent was obtained from the patient for publication of this case report and any accompanying images. A copy of the written consent is available for review by the Editor-in-Chief of this journal.

## Competing interests

The authors declare that they have no competing interests.

## Authors’ contributions

MSB, MMJA and FRC diagnosed the case and supervised the management and follow-up of the patient. MSM and JAQ also involved with managing the case. MMR and FRC wrote the case report. BB also contributed to the writing of the case report. All authors read and approved the final manuscript.
